# Modern Tools of Traditional Oriental Medicine

**DOI:** 10.1155/2019/1642739

**Published:** 2019-02-03

**Authors:** Gihyun Lee, Kyung-Hwa Jung, Sang-Hoon Shin, Hanbing Li

**Affiliations:** ^1^Dongshin University, Naju, Republic of Korea; ^2^University of Pittsburgh, Pittsburgh, PA, USA; ^3^Sangji University, Wonju, Republic of Korea; ^4^Zhejiang University of Technology, Hangzhou, China

Decades ago, Oriental Medicine doctors could use just traditional tools including acupuncture, moxibustion, and herbal medicine despite its long history. However, technology improves and modern medical devices are invented to assist the Oriental Medicine doctors in performing diagnosis and treatment. Now, they can use exclusive medical devices such as pulse meter, tongue diagnosis, and face system with their own sensors and diagnostic skills to get information of body and disease from patients. They also can use modern therapeutic tools including electroacupuncture, pharmacopuncture, and electric-moxibustion as well as traditional ones. Experimental and translational studies on these new tools are underway.

For this special issue, we invited manuscripts with the following topics: improvement of tools in oriental medicine, pharmacopuncture (Chinese medicine injection), electroacupuncture and electric-moxibustion, validation of treatment efficacy, development of pulse meter and tongue diagnosis system, and experimental and translational studies on modern device of Oriental Traditional Medicine. We received 105 manuscripts from various labs for six months and 22 manuscripts were accepted for publication. Here we highlight some of the key ongoing challenges published in this special issue.

The efficacy of medical devices using radiofrequency is demonstrated in the papers “Moxibustion-Simulating Bipolar Radiofrequency Suppresses Weight Gain and Induces Adipose Tissue Browning via Activation of UCP1 and FGF21 in a Mouse Model of Diet-Induced Obesity” and “Short-Term Efficacy of Pulsed Radiofrequency Thermal Stimulation on Acupoints for Chronic Low Back Pain: A Preliminary Study of a Randomized, Single-Blinded, Placebo-Controlled Trial”.

Granule, one of modern forms of herbal medicine, is used in the studies “Study of the Treatment Effects of Compound Tufuling Granules in Hyperuricemic Rats Using Serum Metabolomics” and “Randomized, Double-Blind, Placebo-Controlled Study of Modified Erzhi Granules in the Treatment of Menopause-Related Vulvovaginal Atrophy”.

The article “Cinobufacini Injection Improves the Efficacy of Chemotherapy on Advanced Stage Gastric Cancer: A Systemic Review and Meta-Analysis” reviews the literature on the efficacy comparison between Cinobufacini injection (one of Chinese medicine injections) combined with chemotherapy and chemotherapy solely used in advanced gastric cancer treatment.

The protective effect of patches on acupoints against electromagnetic fields is shown in “Evaluation of the Effectiveness of Protective Patches on Acupoints to Preserve the Bioenergetic Status against Magnetic Fields”.

The clinical application of the low voltage Meridian Energy Detection System is evaluated in assessing the electrodermal activity in the “The Development and Application Evaluation of Meridian Energy Detection System in Traditional Oriental Medicine: A Preliminary Study” and the safety of auto manipulation device for acupuncture which can help Oriental Medicine doctors is shown in the “Safety Assessment of the Auto Manipulation Device for Acupuncture in Sprague-Dawley Rats: Preclinical Evaluation of the Prototype”.

Different face diagnosis system is used in “An Herbal Medicine, Yukgunja-Tang is more Effective in a Type of Functional Dyspepsia Categorized by Facial Shape Diagnosis: A Placebo-Controlled, Double-Blind, Randomized Trial” and “Difference between Right and Left Facial Surface Electromyography in Healthy People”.

In this special issue, there are more valuable manuscripts besides above. We hope the readers will be interested in improvement of diagnostic and remedial tools of Oriental Medicine.

